# Impact evaluation of long-lasting insecticidal nets distribution campaign on malaria cases reported at outpatient departments across all the regions in Ghana

**DOI:** 10.1186/s12936-022-04393-2

**Published:** 2022-12-04

**Authors:** Seth Kwaku Afagbedzi, Yakubu Alhassan, Chris Guure

**Affiliations:** Department of Biostatistics, School of Public Health, University of Ghana, Legon, Accra, Ghana

**Keywords:** Malaria, Long-lasting insecticidal nets, Insecticide-treated nets, Health facilities, Outpatient departments, Ghana

## Abstract

**Background:**

Malaria remains the biggest public health challenge globally, and Ghana is among the 15 highest burden malaria countries in the world, with 2% of global malaria cases and 3% deaths in 2019. This study sought to assess the impact of mass LLIN distribution campaign on malaria morbidity using all health facilities outpatient data across 15 regions of Ghana.

**Methods:**

Data for this study was obtained from the DHIMS2 for health facilities providing OPD and malaria services in Ghana. This was an ecological study that employed the difference-in-difference approach to assess the change in proportion of uncomplicated confirmed malaria cases among OPDs in all types of health facilities in Ghana between 2015 and 2019 following the mass distribution campaign of LLINs in 2018. Malaria cases at the OPDs before and after the free LLIN mass distribution exercise were evaluated.

**Results:**

The trend of the proportion of OPD cases that were confirmed uncomplicated malaria cases increased from 14.8% in 2015 to 18.9% in 2019 at the national level. The average proportion of malaria cases among OPDs in Ghana in 2019 reduced against the expected by − 3.76% (95% CI − 4.69 to − 2.84], p<0.001) among all cases, − 4.52% (95% CI [− 5.70 to − 3.34], p<0.001) among children under-fives years, − 4.10% (95% CI − 5.3 to 2.9], p<0.001) among female children under-five and − 5.18% (95% CI [− 6.33 to − 4.02], p<0.001) among male children under-five. The reduction on the average proportion of malaria cases among OPDs varied significantly across regions and the type of health facilities.

**Conclusion:**

The mass distribution of LLINs across Ghana in 2018 can be associated with reduction in the proportion of malaria cases among OPDs across health facilities in Ghana. The study recommends the biannual mass distribution campaigns especially in the high-density regions.

## Background

Malaria is endemic and perennial in Ghana, with pronounced seasonal variations in the northern part of the country. Ghana is among the 15 highest burden malaria countries in the world, with 2% of global malaria cases and 3% deaths in 2019 [1, 2]. Ghana reported 500,000 more cases from 2017 to 2018, the highest increase in absolute case numbers, representing a 5% increase versus 2017 levels [1]. In 2019, the country recorded approximately 6.7 million presumed and confirmed malaria cases with 336 reported deaths [1, 2]. However, Ghana has made significant progress in malaria control between 2016 and 2019. Number of cases decreased by 32% (from 237 cases per 1000 to 161 cases per 1000 of the population at risk), and deaths decreased 7% (from 0.4 per 1000 to 0.37 per 1000 of the population at risk) [1]. Malaria cases accounted for about 34% of OPD cases, pregnant women constituted 3.9% of the total malaria cases at the OPD [3]. According to National Malaria Control Programme (NMCP), malaria burden is not felt only in the health sector, but in every aspect of the social and economic life of the Ghanaian people, therefore, every effort is being expended for its control and eradication [3].

The distribution of long-lasting insecticidal nets (LLINs) has been a key malaria prevention and control strategy in Ghana for many years. Until recently, this strategy was implemented mainly through social marketing using vouchers, discounted LLIN sales at health facilities, and distribution through measles campaigns. Long-lasting insecticidal nets is a mosquito net saturated with insecticide designed to block mosquitoes physically and for the purpose of killing and repelling mosquitoes which carry malaria parasites [4].

The initial LLIN programme in Ghana targeted the two population groups most likely to suffer severe consequences or die from malaria: children under 5 years of age and pregnant women. The combined strategy of social marketing led to a significant improvement in net coverage: the percentage of children under 5 and pregnant women sleeping under LLIN rose from 4% in 2003 to 28% in 2008 [5]. After 5 years of promoting this strategy, LLIN coverage levelled off at about 28% of children under 5 years and 20% of pregnant women, respectively. Ghana’s NMCP and its partners achieved universal coverage with LLIN through a nationwide door to door distribution and hang up campaign from 2010 to 2012. The objective of universal coverage was to ensure that all members of the population sleep under an LLIN regardless of age or sex. The ultimate goal being that LLIN will protect at least 80% of the population at risk with effective malaria prevention [6, 7]. The use of LLIN is to help halt the process of transmission by eliminating the human reservoir of parasites [8]. In 2013, to sustain universal LLIN coverage, the NMCP initiated a mixed model of LLIN distribution. Alongside with continuous distribution mechanisms using antenatal care, child welfare clinics, schools, shops and workplace programmes, and mass distribution of free LLINs every three years since 2012 [9]. The NMCP distributed about 12.5 million LLINs through a universal mass distribution campaign with hang-up activities in all the then ten regions of Ghana between 2010 and 2012 [10, 11].

To ensure that all households remain protected NMCP in 2013 adopted a Strategic Plan 2014–2020 which aims to protect at least 80 percent of the population at risk with effective malaria prevention interventions by 2020. During the first half of 2016, the PMD of LLIN was conducted in Upper West and Northern regions and a total of 2,457,872 LLINs were distributed [10]. The third round of mass distribution of LLINs ended in December 2018 with about 15.5 million LLINs distributed in 194 districts in nine out of the country’s 10 regions. The most recent mass distributions in Ghana were in 2021. The goal of Ghana’s free mass LLIN distribution campaign was to achieve coverage of at least 90 percent of the general population, providing national access to and use of LLINs [11]. There is strong evidence that when large numbers of people use LLINs to protect themselves while sleeping, the burden of malaria can be reduced, resulting in a reduction in child morbidity and mortality among other benefits [12].

The main objective of this study is to assess the impact of free LLIN distribution campaign on malaria morbidity at the outpatient departments of the health facilities across the 16 regions of Ghana. This is to establish whether the rate of malaria morbidity has increased or decreased in the communities when the NMCP conducted the free point mass distribution of LLIN in 2018 through the universal coverage strategy.

## Methods

### Study design and source of data

This study was an ecological descriptive and exploratory difference-in-difference analysis that used health facility data from the Ghana district health information management system II (DHIMS2). Data from health facilities across all 16 regions in Ghana were used for analysis. The data used in this study consists of health facility visits or Outpatient Department (OPD) data and confirmed complicated malaria cases in the OPDs. The OPD cases in the health facilities served as the denominator whilst the number of confirmed uncomplicated malaria cases were the numerator. Complicated malaria cases were not included in the numerator because such cases are confirmed and treated in the in-patient departments. Both the OPD cases and uncomplicated confirmed malaria cases datasets were extracted from the DHIMS2 database as yearly statistics for each facility across all the 261 districts in the 16 regions in Ghana. Data was extracted for the period 2015 to 2019.

### The long-lasting insecticidal nets point mass distribution (PMD) campaign

The Ghana Malaria Strategic plan (2014–2020) aimed to distribute LLIN across the country to ensure that at least 80% of the population at risk have access to effective malaria prevention interventions. The PMD campaigned aimed to register at least 90% of all households in the targeted regions and distributed LLIN to at least 90% of all the registered households with the universal coverage policy of 2 persons to one LLINs in each household in mind [13]. The PMD exercise excluded the Upper West region and some selected districts in the then Northern region (Now separated into three regions namely Northern, North-East and Savannah regions) and the Ashanti region that had the Indoor Residual Spraying programme that currently on going in 2018 [12, 13].

Prior to the 2018 PMD campaign, the mass distribution of LLINs were conducted in one region at a time. In the 2018 PMD campaign, the distribution was conducted in two phases. Phase I concentrated on distributing data in five regions whilst phase 2 distributed in four other regions. During the campaign an estimated 15,483,708 LLINs was distributed in 194 districts in nine of the 10 regions in the country achieving a national universal coverage of 89% of 1 LLINS for every 2 persons [7]. The was a 99% achievement ratio of the targeted 90%. The LLINs distributed during the campaign is expected to be effective for 3 years.

### Outcome variables

The study estimated the impact of the PMD campaign with focus on four indicators as outcomes which were:

1. The percentage of uncomplicated malaria confirmed cases among all OPD cases across all health facilities per year
2. The percentage of uncomplicated malaria confirmed cases among OPD cases for children under-five years across all health facilities per year
3. The percentage of uncomplicated malaria cases among OPD cases for female children under-5 years across all health facilities per year
4. The percentage of uncomplicated malaria cases among OPD cases for male children under-5 years across all health facilities per year
5. Malaria cases were confirmed using the rapid diagnostic testing (RDT) or the microscopy testing or both.

### Independent variables

Two independent variables namely region and type of health facility were considered for this study.

### Statistical analysis

The number of facilities for each of the 5 years were described using percentages and frequencies. The line plot was used to depict both the expected and observed median of each of the four indicators across the 5 year period. Quantile regression model with robust standard error estimates was used to estimate the median impact of the mass LLINs distribution on each of the four indicators. Sub-groups impact estimates for each of the four indicators were performed at the regional level and type of facility. The difference-in-difference approach was used to assess the impact of the PMD of LLINs on malaria cases in OPDs in Ghana. For each health facility and for each of the four indicators, the number of malaria cases was expressed as a percentage of OPD for each of the 5 years. The expected difference in each of the four indicators between 2017 and 2019 were obtained by finding the median of the differences between the percentage of malaria cases in OPDs between 2015 and 2017 for each of the health facilities. Thus:

$${E}_{i, \left(2017 to 2019\right)}=median\left({P}_{i, 2017, k}-{P}_{i,2015,k}\right)$$

Where, ${P}_{i,j,k}$ is the percentage of malaria cases for indicator $i$ year $j$ and facility $k$, ${E}_{i,\left(2017 to 2019\right)}$ is the expected difference of malaria cases for indicator i between 2017 and 2019

The observed difference for each health facility for each of the four indicators were then obtained by finding the difference between the percentage of malaria cases in OPDs between 2017 and 2019 for each health facility.

Thus:

$${O}_{i, \left(2017 to 2019\right)}=median\left({P}_{i, 2019, k}-{P}_{i,2017,k}\right)$$

where ${O}_{i, \left(2017 to 2019\right)}$ is the observed difference of malaria cases for indicator $i$ between 2017 and 2019.

The impact of the mass LLINs distribution was estimated as the median of a difference-in-difference (DID). The DID was computed as the difference between the observed and the expected for each health facility for each of the four indicators. Thus:

$$DI{D}_{i, 2017 to 2019, k}={O}_{i,\left(2017 to 2019\right),k}-{E}_{i,\left(2017 to 2019\right), k}$$

The impact for each indicator was then estimated as the median of the DIDs for each indicator. Thus

$$Impac{t}_{estimat{e}_{i}} =median \left(DI{D}_{i,\left(2017 to 2019\right),k}\right)$$

Impact was estimated at the national level, regional level and also by facility type. All statistical analyses were performed using Stata IC version 16 (Stata Corp., College Station, TX, USA). All statistical analysis was considered significant at 0.05 alpha level.

## Results

### Number of health facilities by year and region

In 2015, 3650 different facilities were considered, this number increased to 3931 in 2016. In the year 2017, the total number of the different facilities was 4323 followed by, 4434 in 2018 and then 4769 in 2019. In 2019, the Eastern (14.5%) and the Ashanti (11.2%) regions had the highest number of health facilities among the 16 regions. Most of the health facilities were Community-based Health Planning and Services (CHPS) compounds (Table 1).

**Table 1:** Number of health facilities for each year by regions, facility type and level of facility

	Years				
	2015	2016	2017	2018	2019
	N = 3650	N = 3931	N = 4323	N = 4434	N = 4769
Region					
Ahafo	63 (1.7)	67 (1.7)	73 (1.7)	79 (1.8)	80 (1.7)
Ashanti	411 (11.3)	451 (11.5)	482 (11.1)	497 (11.2)	535 (11.2)
Bono	179 (4.9)	183 (4.7)	194 (4.5)	201 (4.5)	205 (4.3)
Bono east	132 (3.6)	137 (3.5)	146 (3.4)	148 (3.3)	159 (3.3)
Central	355 (9.7)	371 (9.4)	409 (9.5)	423 (9.5)	463 (9.7)
Eastern	551 (15.1)	579 (14.7)	642 (14.9)	638 (14.4)	690 (14.5)
Greater accra	263 (7.2)	279 (7.1)	314 (7.3)	310 (7.0)	356 (7.5)
North-East	54 (1.5)	55 (1.4)	60 (1.4)	64 (1.4)	66 (1.4)
Northern	171 (4.7)	185 (4.7)	236 (5.5)	254 (5.7)	263 (5.5)
Oti	125 (3.4)	131 (3.3)	142 (3.3)	152 (3.4)	161 (3.4)
Savannah	87 (2.4)	96 (2.4)	101 (2.3)	109 (2.5)	114 (2.4)
Upper East	278 (7.6)	290 (7.4)	297 (6.9)	303 (6.8)	357 (7.5)
Upper West	249 (6.8)	292 (7.4)	314 (7.3)	324 (7.3)	338 (7.1)
Volta	272 (7.5)	289 (7.4)	309 (7.1)	327 (7.4)	333 (7.0)
Western	290 (7.9)	350 (8.9)	392 (9.1)	385 (8.7)	412 (8.6)
Western north	170 (4.7)	176 (4.5)	212 (4.9)	220 (5.0)	237 (5.0)
Facility type					
Hospital	356 (9.8)	369 (9.4)	388 (9.0)	398 (9.0)	434 (9.1)
Polyclinic	47 (1.3)	47 (1.2)	48 (1.1)	47 (1.1)	56 (1.2)
Medical centre	37 (1.0)	42 (1.1)	52 (1.2)	52 (1.2)	70 (1.5)
Health centre	920 (25.2)	942 (24.0)	953 (22.0)	960 (21.7)	979 (20.5)
Maternity home	155 (4.2)	163 (4.1)	167 (3.9)	163 (3.7)	158 (3.3)
Clinic	456 (12.5)	475 (12.1)	508 (11.8)	503 (11.3)	534 (11.2)
CHPS	1679 (46.0)	1893 (48.2)	2207 (51.1)	2311 (52.1)	2538 (53.2)

### Proportion of confirmed uncomplicated malaria cases in OPD at national and regional level

**Fig. 1:**
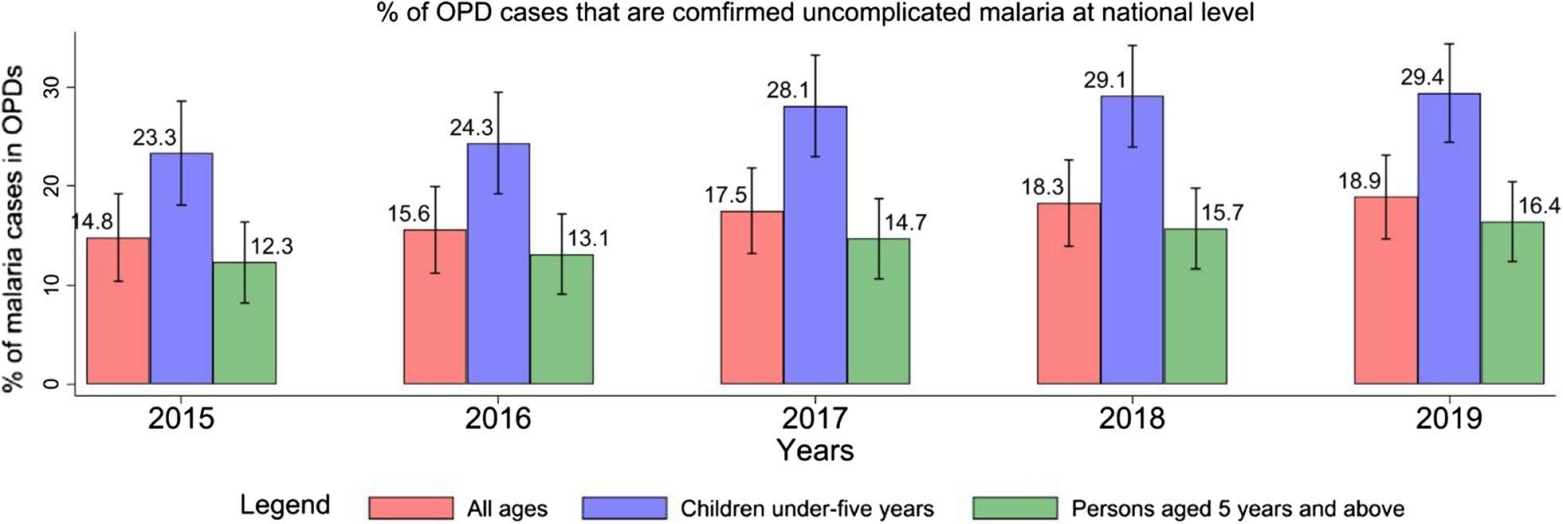
The trend of the percentage of OPDs cases that are confirmed uncomplicated malaria cases in at the national level.

At the national level, the proportion of confirmed uncomplicated malaria cases among all OPD cases was 14.8% in 2015 which steadily increased annually to 18.9% in 2019. Among children under-5 years, the percentage of OPD cases that were confirmed uncomplicated malaria cases steadily increased from 23.3% in 2015 to 29.4% in 2019. Also, among persons aged 5 years and above visiting the OPDs, the percentage of confirmed uncomplicated malaria cases also steadily increased from 12.3% in 2015 to 16.4% in 2019 (Fig. 1).

**Fig. 2:**
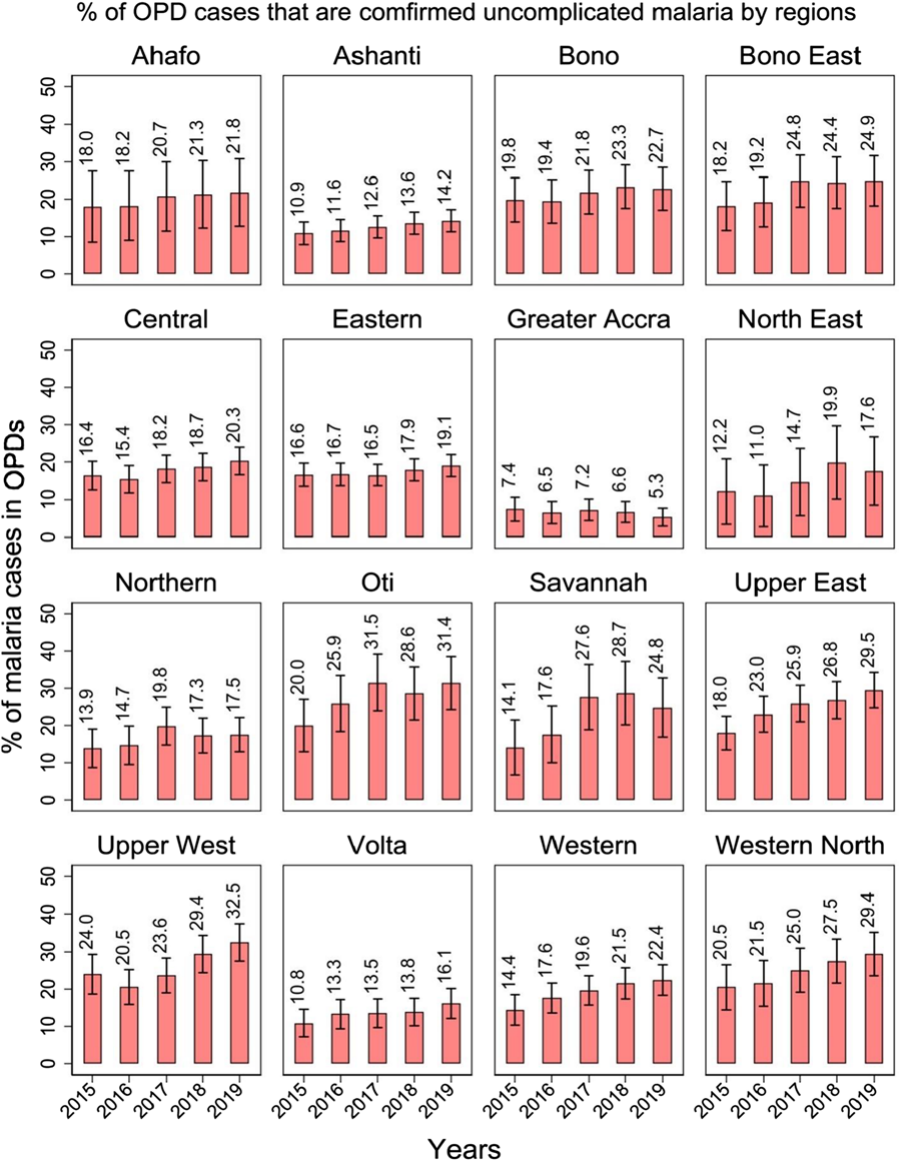
The trend of the percentage of OPDs cases that are confirmed uncomplicated malaria cases by region from 2015 to 2019

Figure 2 shows the percentage trend of malaria cases among all OPD cases at regional levels. During the 5 year period starting from 2015 and then to 2019, the proportion of OPD malaria cases increased from 18.0% to 21.8% in Ahafo, 10.9% to 14.2% in Ashanti, 19.8% to 22.7% in Bono, 18.2% to 24.9% in Bono East, 16.4% to 20.3% in Central, 16.6% to 19.1% in Eastern, 12.2% to 17.6% in North East, 13.9% to 17.5% in Northern, 20.0% to 31.4% in Oti, 14.1% to 24.8% in Savannah, 18.0% to 29.5% in Upper East, 24.0% to 32.5% in Upper West, 10.8% to 16.1% in Volta, 14.4% to 22.4% in Western and 20.5% to 29.4% in Western North. Greater Accra was the only region to have recorded a decrease in malaria cases from 7.4% to 5.3% (Fig. 2).

### The trends of the expected and observed percentage of malaria cases in OPDs in Ghana from 2015 to 2019

**Fig. 3:**
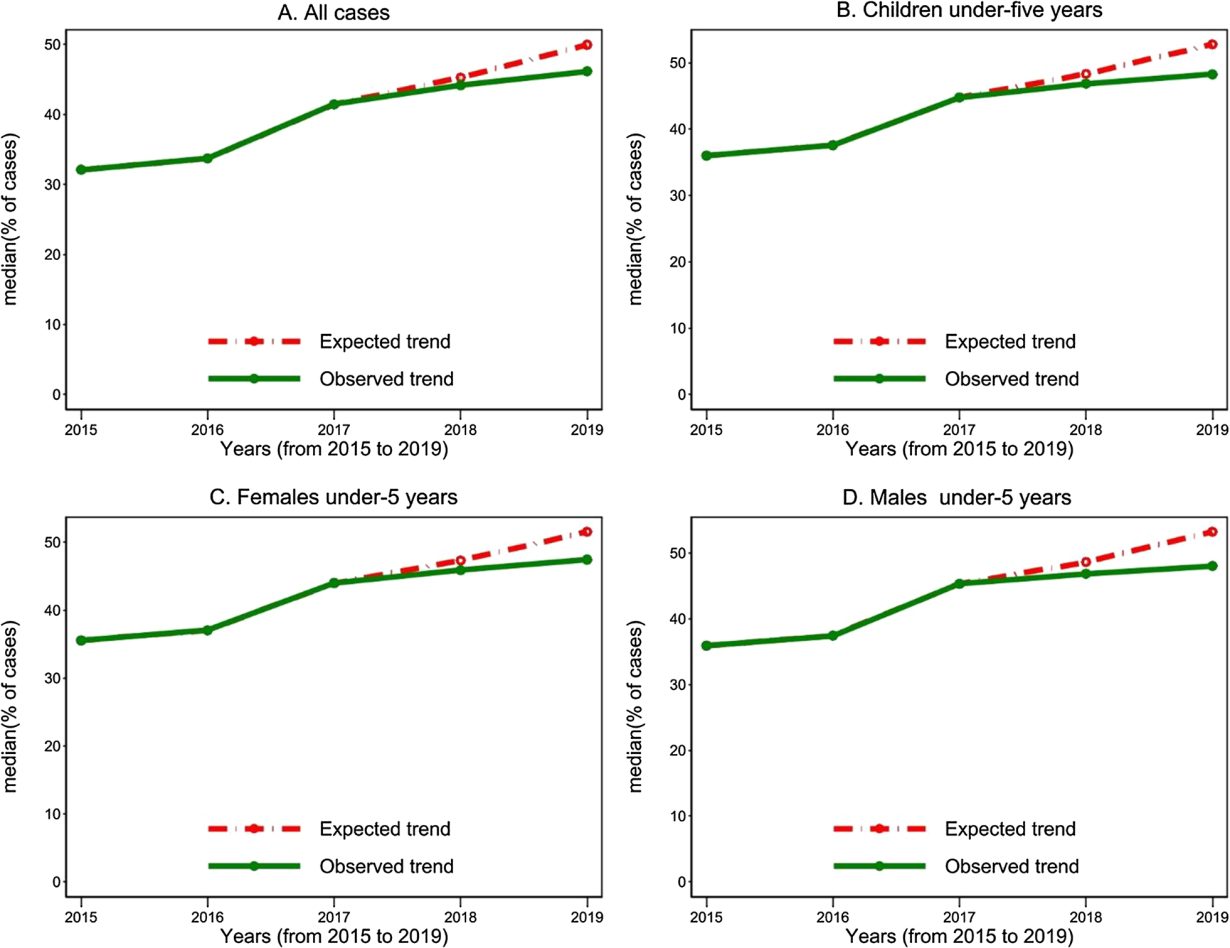
Trends of expected and observed percentages of malaria cases among OPD cases, for all cases, Female cases, Male cases, and Under-5 cases

The average percentage of total uncomplicated malaria cases among all OPD cases in Ghana saw an upward trajectory from 2015 to 2019, however the rate of increase slowed between the period 2017 and 2019. Similar trend was observed for the proportion of uncomplicated malaria cases among OPDs for all children under five years as well as children under-5 years disaggregated by sex (Fig. 3). The general increase in OPD malaria cases can be observed for the period under review by region but with a very moderate increase towards 2017 to 2019, for most regions. In 2015, the average percentage of uncomplicated malaria cases among OPD cases was 32.12% (95% CI: [31.2–33.1]) which increased to 33.7% (95% CI [32.7–34.7]) in 2016, and then to 46.18% (95% CI [45.3–47.1]) in 2019 (Table 2). Further information on cases for children under-five and by sex for the 5 years is provided (Table 2).

**Table 2:** The impact of PMD of LLINs on the number of OPD reported malaria cases as a percentage of all OPD cases in Ghana

Level of impact estimate	Impact estimates of PMD of LLINs on the percentage malaria morbidity in OPDS in Ghana			
	All age groups	Under-five (All)	Under-five (Females)	Under-five (Males)
	Median (95% CI)	Median (95% CI)	Median (95% CI)	Median (95% CI)
National estimate				
Observed				
2015	32.12 [31.16, 33.07]	36.02 [34.89, 37.16]	35.56 [34.40, 36.71]	35.93 [34.81, 37.05]
2016	33.72 [32.74, 34.70]	37.57 [36.46, 38.68]	37.04 [35.88, 38.19]	37.47 [36.33, 38.62]
2017	41.43 [40.44, 42.43]	44.77 [43.57, 45.97]	44.00 [42.84, 45.16]	45.33 [44.07, 46.58]
2018	44.18 [43.21, 45.15]	46.85 [45.74, 47.95]	45.89 [44.77, 47.01]	46.86 [45.74, 47.99]
2019	46.18 [45.25, 47.10]	48.24 [47.06, 49.43]	47.44 [46.29, 48.58]	48.05 [46.90, 49.20]
Expected (2019)	49.94 [47.68, 52.21]	52.76 [50.21, 55.31]	51.54 [49.00, 54.07]	53.23 [50.50, 55.95]
Impact estimate (2019)	− 3.76 [− 4.69, − 2.84]***	− 4.52 [− 5.70, − 3.34]***	− 4.10 [− 5.25, − 2.95]***	− 5.18 [− 6.33, − 4.02]***
National				
Regional				
Ahafo	− 5.25 [− 15.73, 5.22]	− 7.65 [− 19.32, 4.03]	− 7.44 [− 18.98, 4.10]	− 5.33 [− 18.21, 7.54]
Ashanti	5.49 [2.00, 8.99]**	3.52 [− 0.38, 7.41]	3.87 [− 0.07, 7.80]	3.34 [− 0.70, 7.39]
Bono	− 4.83 [− 8.34, − 1.33]**	− 6.93 [− 11.00, − 2.85]**	− 5.54 [− 9.45, − 1.62]**	− 5.56 [− 9.72, − 1.41]**
Bono east	− 4.47 [− 8.37, − 0.58]*	− 2.89 [− 7.15, 1.38]	− 5.04 [− 9.28, − 0.79] *	− 4.22 [− 8.86, 0.41]
Central	− 3.69 [− 6.29, − 1.08]**	− 7.36 [− 10.26, − 4.46]***	− 6.14 [− 9.21, − 3.06]***	− 4.34 [− 7.66, − 1.03]*
Eastern	3.70 [1.42, 5.98]**	3.42 [0.98, 5.86]**	3.05 [0.61, 5.49]*	3.56 [1.12, 6.01]**
Greater accra	− 0.67 [− 2.73, 1.39]	− 3.03 [− 5.23, − 0.82]**	− 0.76 [− 2.75, 1.23]	− 1.67 [− 4.04, 0.70]
North-East	7.09 [− 2.93, 17.12]	7.88 [− 1.53, 17.28]	3.16 [− 6.33, 12.65]	7.20 [− 2.26, 16.65]
Northern	− 27.62 [− 31.93, − 23.31]***	− 33.54 [− 38.70, − 28.37]***	− 37.22 [− 42.02, − 32.41]***	− 37.30 [− 42.60, − 32.01]***
Oti	− 18.21 [− 23.30, − 13.11]***	− 21.47 [− 25.95, − 16.99]***	− 20.95 [− 26.22, − 15.68]***	− 23.04 [− 27.21, − 18.87]***
Savannah	− 26.15 [− 31.76, − 20.53]***	− 31.80 [− 38.41, − 25.20]***	− 30.15 [− 36.41, − 23.89]***	− 28.75 [− 35.03, − 22.48]***
Upper East	− 9.11 [− 11.28, − 6.94]***	− 7.03 [− 9.53, − 4.53]***	− 7.88 [− 9.87, − 5.88]***	− 8.78 [− 11.34, − 6.21]***
Upper West	6.69 [4.49, 8.89]***	1.97 [− 0.60, 4.54]	3.93 [1.12, 6.75]**	5.50 [3.04, 7.95]***
Volta	− 6.33 [− 9.39, − 3.27]***	− 6.04 [− 9.73, − 2.34]**	− 2.71 [− 6.49, 1.07]	− 5.60 [− 9.31, − 1.89]**
Western	− 6.89 [− 10.71, − 3.06]***	− 8.59 [− 12.64, − 4.54]***	− 6.58 [− 10.75, − 2.40]**	− 8.59 [− 12.50, − 4.69]***
Western north	1.82 [− 1.99, 5.64]	− 4.00 [− 7.67, − 0.34]*	− 9.36 [− 13.36, − 5.35]***	− 5.00 [− 8.90, − 1.10]*
Facility type				
Hospital	− 0.64 [− 1.54, 0.25]	− 2.63 [− 4.05, − 1.21]***	− 1.66 [− 3.10, − 0.21]*	− 1.92 [− 3.35, − 0.48]**
Polyclinic	− 1.82 [− 7.69, 4.05]	− 2.29 [− 10.02, 5.43]	− 1.07 [− 9.09, 6.95]	− 2.95 [− 10.86, 4.96]
Medical centre	− 2.70 [− 6.65, 1.26]	− 7.69 [− 12.64, − 2.74]**	− 4.91 [− 10.27, 0.44]	− 4.85 [− 9.50, − 0.20]*
Health centre	0.95 [− 0.40, 2.29]	− 1.90 [− 3.77, − 0.02]*	− 1.68 [− 3.52, 0.16]	− 3.20 [− 5.08, − 1.33]**
Maternity home	4.46 [0.42, 8.50] *	− 4.08 [− 9.18, 1.02]	− 3.44 [− 8.25, 1.37]	− 1.81 [− 7.54, 3.91]
Clinic	0.05 [− 2.05, 2.16]	0.00 [− 2.83, 2.83]	0.00 [− 2.94, 2.94]	0.00 [− 2.96, 2.96]
CHPS	0.00 [− 0.94, 0.94]	0.00 [− 1.25, 1.25]	0.00 [− 1.28, 1.28]	− 0.01 [− 1.31, 1.30]

P-value notation

^*^p<0.001

^**^p<0.01

^*^p<0.05

### The impact of PMD of LLINs on the percentage of OPD registrants confirmed of having malaria between 2017 and 2019

The percentage of total OPD malaria cases was expected to rise to 49.94% (95% CI [47.7 to 52.2]) in 2019 from 2017, however, the observed rise was 46.18% (95% CI [45.25 to 47.10]) which was significantly less than the expected by -3.76% (95% CI − 4.69 to− 2.84], p<0.001). Among children under-5 years, it was expected to rise to 52.76% (95% CI [50.2 to 55.3]) in 2019 from 2017, but rather, the observed rise was 48.24% (95% CI [47.1 to 49.4]) with a statistical significance reduction of − 4.52% (95% CI [− 5.70 to − 3.34], p<0.001) (Table 2).

Further disaggregating the data for children under-5 years by sex, the median of percentage of OPD cases that were confirmed uncomplicated malaria among females’ children was observed to be 47.44% (95% CI [46.3–48.6]) as against an expected rise of 51.54% (95% CI [49.0–54.1]) in 2019 which was significantly less by − 4.10% (95% CI − 5.3 to 2.9], p<0.001). Also, the median percentage of OPD cases that were confirmed uncomplicated malaria among the male children was observed to be 48.05% (95% CI [46.9 to 49.2]) against an expected rise of 53.23% (95% CI [50.50–55.95]) which with a significantly less value than the expected by − 5.18% (95% CI [− 6.33 to − 4.02], p<0.001) (Table 2).

### Regional, type of facility and facility level analyses of the impact of PMD of LLINs on proportion of malaria cases in OPD attendants in Ghana between 2017 and 2019

#### Uncomplicated confirmed malaria among all OPD cases

The median percentage of OPD cases that were confirmed uncomplicated malaria cases did not see significant changes in 4 of the 16 regions which included Ahafo, Greater Accra, North-East and Western North regions. There were significant reduction in the median percentage of OPD cases that were confirmed uncomplicated malaria cases among children under-5 years in 9 regions namely the Bono (− 4.83, 95% CI − 8.34 to − 1.33]), Bono East (− 4.47, 95% CI − 8.37 to − 0.58]), Central (− 3.69, 95% CI − 6.29 to − 1.08]), Northern (− 27.62, 95% CI − 31.93 to − 23.31]), Oti (− 18.21, 95% CI − 23.30, − 13.11]), Savannah (− 26.15, 95% CI [− 31.76 to − 20.53]), Upper East (− 9.11, 95% CI [− 11.28 to − 6.94]), Volta (− 6.33, 95% CI − 9.39, − 3.27]) and Western (− 6.89, 95% CI [− 10.71 to − 3.06]). On the other, significant increase in the median percentage of OPD cases that were confirmed uncomplicated malaria cases in 3 regions namely Ashanti (5.49, 95% CI [2.00 to 8.99]), Eastern (3.70, 95% CI [1.42 to 5.98]) and the Upper West (6.69, 95% CI: [4.49 to 8.89]) (Table 3).

**Table 3:** Impact estimates of PMD of LLINs on the percentage malaria morbidity in OPDS in Ghana

		Children under-5 years		
Level of impact estimate	All cases	Under-five	Female cases	Male cases
	Median (95% CI)	Median (95% CI)	Median (95% CI)	Median (95% CI)
National	− 3.77 [− 4.69, − 2.84]^c^	− 4.52 [− 5.70, − 3.34]c	− 4.10 [− 5.25, − 2.95]c	− 5.17 [− 6.33, − 4.02]c
Region				
Ahafo	− 5.25 [− 15.73, 5.22]	− 7.65 [− 19.32, 4.03]	− 7.44 [− 18.98, 4.10]	− 5.33 [− 18.21, 7.54]
Ashanti	5.49 [2.00, 8.99]b	3.52 [− 0.38, 7.41]	3.87 [− 0.07, 7.80]	3.34 [− 0.70, 7.39]
Bono	− 4.83 [− 8.34, − 1.33]^b^	− 6.93 [− 11.00, − 2.85]b	− 5.54 [− 9.45, − 1.62]b	− 5.56 [− 9.72, − 1.41]^b^
Bono East	− 4.47 [− 8.37, − 0.58]^a^	− 2.89 [− 7.15, 1.38]	− 5.04 [− 9.28, − 0.79]a	− 4.22 [− 8.86, 0.41]
Central	− 3.69 [− 6.29, − 1.08]^b^	− 7.36 [− 10.26, − 4.46]c	− 6.14 [− 9.21, − 3.06]c	− 4.34 [− 7.66, − 1.03]a
Eastern	3.70 [1.42, 5.98]^b^	3.42 [0.98, 5.86]b	3.05 [0.61, 5.49]a	3.56 [1.12, 6.01]b
Greater accra	− 0.67 [− 2.73, 1.39]	− 3.03 [− 5.23, − 0.82]b	− 0.76 [− 2.75, 1.23]	− 1.67 [− 4.04, 0.70]
North-East	7.09 [− 2.93, 17.12]	7.88 [− 1.53, 17.28]	3.16 [− 6.33, 12.65]	7.20 [− 2.26, 16.65]
Northern	− 27.62 [− 31.93, − 23.31]^c^	− 33.54 [− 38.70, − 28.37]^c^	− 37.22 [− 42.02, − 32.41] c	− 37.30 [− 42.60, − 32.01]^c^
Oti	− 18.21 [− 23.30, − 13.11]^c^	− 21.47 [− 25.95, − 16.99]^c^	− 20.95 [− 26.22, − 15.68]c	− 23.04 [− 27.21, − 18.87]^c^
Savannah	− 26.15 [− 31.76, − 20.53]^c^	− 31.80 [− 38.41, − 25.20]^c^	− 30.15 [− 36.41, − 23.89]^c^	− 28.75 [− 35.03, − 22.48]^c^
Upper east	− 9.11 [− 11.28, − 6.94]^c^	− 7.03 [− 9.53, − 4.53c	− 7.88 [− 9.87, − 5.88]^c^	− 8.78 [− 11.34, − 6.21]^c^
Upper west	6.69 [4.49, 8.89]^c^	1.97 [− 0.60, 4.54]	3.93 [1.12, 6.75]^b^	5.50 [3.04, 7.95]^c^
Volta	− 6.33 [− 9.39, − 3.27]^c^	− 6.04 [− 9.73, − 2.34]^b^	− 2.71 [− 6.49, 1.07]	− 5.60 [− 9.31, − 1.89]^b^
Western	− 6.89 [− 10.71, − 3.06]^c^	− 8.59 [− 12.64, − 4.54]^c^	− 6.58 [− 10.75, − 2.40]^b^	− 8.59 [− 12.50, − 4.69]^c^
Western north	1.82 [− 1.99, 5.64]	− 4.00 [− 7.67, − 0.34]^a^	− 9.36 [− 13.36, − 5.35]^c^	− 5.00 [− 8.90, − 1.10]^a^
Facility type				
Hospital	− 0.64 [− 1.54, 0.25]	− 2.63 [− 4.05, − 1.21]^c^	− 1.66 [− 3.10, − 0.21]^a^	− 1.92 [− 3.35, − 0.48]^b^
Polyclinic	− 1.82 [− 7.69, 4.05]	− 2.29 [− 10.02, 5.43]	− 1.07 [− 9.09, 6.95]	− 2.95 [− 10.86, 4.96]
Medical centre	− 2.70 [− 6.65, 1.26]	− 7.69 [− 12.64, − 2.74]^b^	-4.91 [− 10.27, 0.44]	− 4.85 [− 9.50, − 0.20]^a^
Health centre	0.95 [− 0.40, 2.29]	− 1.90 [− 3.77, − 0.02]^a^	− 1.68 [− 3.52, 0.16]	− 3.20 [− 5.08, − 1.33]^b^
Maternity home	4.46 [0.42, 8.50]^a^	− 4.08 [− 9.18, 1.02]	− 3.44 [− 8.25, 1.37]	− 1.81 [− 7.54, 3.91]
Clinic	0.05 [− 2.05, 2.16]	0.00 [− 2.83, 2.83]	0.00 [− 2.94, 2.94]	0.00 [− 2.96, 2.96]
CHPS	0.00 [− 0.94, 0.94]	0.00 [− 1.25, 1.25]	0.00 [− 1.28, 1.28]	− 0.01 [− 1.31, 1.30]

In terms of facility type, the percentage of OPD cases that were confirmed uncomplicated malaria cases did not see significant changes by facility type except for the maternity home (4.46%, 95% CI [0.42–8.50]) were there were significant increased (Table 3).

#### Uncomplicated confirmed malaria among all children under-five years

Among children under-5 years, the median percentage of OPD cases that were confirmed uncomplicated malaria cases did not see significant changes 5 of the 16 regions which included Ahafo, Ashanti, Bono East, North-East, and Upper West regions. There were significant reduction in the median percentage of OPD cases that were confirmed uncomplicated malaria cases in 10 regions namely the Bono (− 6.93, 95% CI [− 11.00 to − 2.85]), Central (− 7.36, 95% CI: [− 10.26 to − 4.46]), Greater Accra (− 3.03, 95% CI [− 5.23 to − 0.82]), Northern (− 33.54, 95% CI [− 38.70 to − 28.37]), Oti (− 21.47, 95% CI [− 25.95 to − 16.99]), Savannah (− 31.80, 95% CI [− 38.41 to − 25.20]), Upper East (− 7.03, 95% CI [− 9.53 to − 4.53]), Volta (− 6.04, 95% CI [− 9.73 to − 2.34]), Western (− 8.59, 95% CI: [− 12.64 to 4.54]) and Western North (− 4.00, 95% CI: [− 7.67 to − 0.34]). On the other, significant increase in the median percentage of OPD cases that were confirmed uncomplicated malaria cases among children under-five years was recorded only in the Eastern region (3.42, 95% CI [0.98 to 5.86]) (Table 3).

In terms of facility type, the percentage of OPD cases that were confirmed uncomplicated malaria cases among children under-five years significantly reduced in the Hospitals (− 2.63, 95% CI [− 4.05 to − 1.21]) the medical centres (− 7.69, 95% CI [− 12.64 to − 2.74]) and the health centres (− 1.90, 95% CI [− 3.77 to − 0.02]) (Table 2). In Table 3 the impact estimated among children under-five years disaggregated by sex was also estimated for female children and male children (Table 3).

## Discussion

This study sought to assess the potential impact on malaria morbidity after free LLIN distribution campaign in 2018. The proportion of OPD cases that were confirmed uncomplicated malaria cases in health facilities across in all 16 regions in Ghana were considered. The point mass distribution of LLINs was conducted in 2018, hence annual health facility data from 2015 through to 2019 was used. LLIN has been identify as a very effective vector control intervention and the cornerstone of malaria prevention in Sub-Saharan Africa [13]. The distribution of LLINs in Ghana is usually through mass campaigns and continuous distribution through antenatal care clinics, child welfare clinics and primary schools targeting pregnant women, children under-five, and school-aged children, respectively [14].

This study revealed a general increase in the trend of proportion of OPD attendants who were diagnosed of malaria from 2015 to 2019. However, findings from this study show the increase in the proportion of malaria cases in OPDs between 2017 and 2019 was lower than expected as estimated from the trend of increase between 2015 and 2017. Among all ages, the median proportion of OPDs cases that were confirmed uncomplicated malaria cases steadily increased to 46.1% as against the expected 49.9%. For children under-5 years, there was an estimated 4.5% decrease in the median proportion OPD cases that were confirmed uncomplicated malaria cases against the expected. In terms of sex disaggregation among children under-5 years, the median proportion was decreased by 4.1% as against the expected 51.5% for females and 5.2% as against the expected 53.2% among males. Further sub-analysis from the current study showed disparities in the reduction of proportion of malaria cases against the expected rise by health facility type.

Comparison from other studies affirm the results reported in the current study. Results from a scale-up of a programme for malaria vector control between 2006 and 2009 in South Sudan showed that, household access to at least one LLIN increased from about 12% to 53% and LLIN utilization rates increased from 5 to 25% among children younger than 5 years and from 5 to 36% among pregnant women. However, the number of recorded malaria cases increased from 71,948 in 2008 to 1,198,357 in 2012 [15]. In Ghana, access to LLINs also showed around 7% absolute reduction in self-reported malaria prevalence among women living in households with access to ITNs [16]. Similarly, the current result is in line with the findings from the World malaria report 2020, which stated that there were fewer malaria cases in 2000 (204 million) than in 2019 (237 million) in the WHO African Region [2], which means there have been year by year increases in malaria infections across the region over the period. However, at the same time, the incidence of malaria reduced from 363 to 225 cases per 1000 population in the region. These statistics demonstrate the complexity of interpreting the changing malaria transmission in a rapidly increasing population in WHO African region where several malaria interventions have and are being implemented aimed at eradicating the disease. The slow rate of increase in the expected proportion of the OPD attendants diagnosed with malaria from 2017 to 2019 from this study may be partly attributed to the continuous ownership and possible use of the LLIN received through PMD campaign.

Although, other malaria prevention and treatment strategies such as continuous LLINs distribution at ANCs, indoor residual spraying in selected regions, Seasonal malaria chemotherapy in some regions [14], the 2018 PMD campaign was a massive action that was carried out in Ghana hence may have contributed significantly to the fight against malaria as the findings of this study suggests. The result from this study demonstrates significant reduction in proportion of OPD cases that were malaria that may be attributed to the mass distribution of LLINS in the country in 2018. Regional level analysis in some regions also showed significant decrease in the median proportion of malaria cases among OPDs in health facilities in 2019 compared to expected. In the Upper West region were the PMD campaign was not conducted, this study did not record significant changes. The Northern region recorded the highest impact for all the four indicators. The impact was not significant in Ahafo, and North East regions for all four indicators.

The variation in the significant reductions in malaria cases from this study is also supported by findings from the malaria indicator survey of 2016 data among women of reproductive age [16]. Also, a study conducted to assess the impact of mass distribution of free long-lasting insecticidal nets on childhood malaria morbidity in Togo National Integrated Child Health Campaign, observed a marked reduction in childhood malaria associated morbidity in the year following mass distribution of free LLINs in two of three districts in Togo [17].

It is reported elsewhere that when full coverage of ITNs is achieved, it can reduce child mortality by 17% in sub-Saharan Africa [4]. The study further stated that ITNs reduce clinical episodes of uncomplicated malaria caused by *Plasmodium falciparum* and *Plasmodium vivax* by 50%, as well as reducing the prevalence of high density parasitaemia. In a related study, it has been demonstrated that protection against forest malaria in the Amazon region and in Cambodia is an important aspect of the action of ITNs [18]. A study examining the effect of LLIN use on malaria prevalence in the Tombel Health District (THD), South-West Region-Cameroon, found that the distribution of LLINs led to a short-lived reduction in malaria prevalence in THD the following year [19]. The results of the study showed that the number of confirmed cases was highest from June to August (peak rainy season) the following year. Malaria prevalence increased in THD from 26.7% in 2010 to 30.7% in 2011 but dropped to 22.7% in 2012. There was an overall drop in the total number of confirmed malaria cases in 2012 in some areas [19].

## Limitations

This study is more of an ecological design and, therefore, conclusion drawn should be interpreted with caution. The study assumed the mass distribution of the LLINs will directly lead to decreased in the number of malaria cases reported in OPDs in all the health facilities in the country, this assumption may not be sacrosanct. Although, households received LLINs, it does not necessarily translate to utilization, though it is expected that households exposed to mosquito bites are more likely to use LLINs once available. This directly leads to reduction in malaria morbidity and subsequently the number of malaria cases reported at OPDs in health facilities. Also, no vigorous statistical modelling could be performed due limited number of variables available in the dataset. The analysis could not control for other potential confounders at the individual, household or community due to limited information and the aggregate nature of the data. Also, the quality of the data used is vastly limited to the level of quality from the DHIMS data. The current study only excluded health facilities that did not report on any OPD attendance.

## Conclusion

The mass distribution of LLINs across Ghana in 2018 contributed to a significant drop in the proportion of malaria cases among OPDs in Ghana. The drops were significant for most of the regions and also across the various types of health facilities. The study recommends the continuous biannual mass distribution campaigns especially in the high-density regions.

## Data Availability

The datasets used and/or analysed during the current study are available from the corresponding author on reasonable request.

## Abbreviations

DHIMS2: District health information management system II
LLIN: Long-lasting insecticidal net
OPD: Outpatients department
PMD: Point mass distribution
NMCP: National malaria control programme
